# Future-Proofing Your *Microbiology Resource Announcements* Genome Assembly for Reproducibility and Clarity

**DOI:** 10.1128/MRA.00954-19

**Published:** 2019-09-05

**Authors:** David A. Baltrus, Christina A. Cuomo, John J. Dennehy, Julie C. Dunning Hotopp, Julia A. Maresca, Irene L. G. Newton, David A. Rasko, Antonis Rokas, Simon Roux, Jason E. Stajich

**Affiliations:** a School of Plant Sciences, University of Arizona, Tucson, Arizona, USA; b Broad Institute of MIT and Harvard, Cambridge, Massachusetts, USA; c Queens College of The City University of New York, Queens, New York, USA; d The Graduate Center of The City University of New York, New York, New York, USA; e Institute for Genome Sciences, University of Maryland Baltimore, Baltimore, Maryland, USA; f Department of Microbiology and Immunology, University of Maryland Baltimore, Baltimore, Maryland, USA; g Greenebaum Comprehensive Cancer Center, University of Maryland Baltimore, Baltimore, Maryland, USA; h Department of Civil and Environmental Engineering, University of Delaware, Newark, Delaware, USA; i Department of Biology, Indiana University, Bloomington, Indiana, USA; j Department of Biological Sciences, Vanderbilt University, Nashville, Tennessee, USA; k Department of Biomedical Informatics, Vanderbilt University, Nashville, Tennessee, USA; l Vanderbilt Genetics Institute, Vanderbilt University Medical Center, Nashville, Tennessee, USA; m DOE Joint Genome Institute, Walnut Creek, California, USA; n Department of Microbiology and Plant Pathology and Institute for Integrative Genome Biology, University of California—Riverside, Riverside, California, USA

## Abstract

Descriptions of resources, like the genome assemblies reported in *Microbiology Resource Announcements*, are often frozen at their time of publication, yet they will need to be interpreted in the midst of continually evolving technologies. It is therefore important to ensure that researchers accessing published resources have access to all of the information required to repeat, interpret, and extend these original analyses. Here, we provide a set of suggestions to help make certain that published resources remain useful and repeatable for the foreseeable future.

## Abstract (precis)

Descriptions of resources, like the genome assemblies reported in *Microbiology Resource Announcements*, are often frozen at their time of publication, yet they will need to be interpreted in the midst of continually evolving technologies. It is therefore important to ensure that researchers accessing published resources have access to all of the information required to repeat, interpret, and extend these original analyses.

## EDITORIAL

There are many ways to sequence and assemble a genome, with the number of available sequencing and assembly platforms seemingly growing every week. Within sequencing platforms, library preparation, chemistry, and error profiles frequently change. Our primary goal as *Microbiology Resource Announcements* (MRA) editors is to ensure that a manuscript’s techniques and protocols are thoroughly documented so that readers can understand the strengths and weaknesses not only of a particular genome assembly but also the underlying raw data. Given the importance of clarity of workflows and reproducibility of data in validating scientific results (1–3), we want to ensure that all of the relevant data contributing to an assembly are available for other researchers so that they can (i) reproduce the study’s results, (ii) elaborate and incorporate the available data into other genome assemblies, or (iii) repurpose public data for use in alternative analyses. While many of these current best practices have been incorporated into the Instructions to Authors, in this opinion piece, we aim to provide a set of thematic ideas and examples behind certain instructions for authors to increase reproducibility across groups and utility for future users. We also highlight the fact that groups have proposed sets of standards for isolate genomes (4), 16S rRNA/18S rRNA/other amplicons (5), and single-cell amplified genomes (SAGs) and metagenome-assembled genomes (MAGs) (6) and that recommendations from those proposals are highly relevant and compatible with points raised in this editorial.

### Strain provenance and culture conditions.

Even before DNA extraction, it is important to document how particular isolates were isolated, cultured, and maintained. When and where was the strain isolated? What was the culture collection source? Has the strain been passaged since its isolation or acquisition from a culture collection? Was a single colony or plaque picked to amplify the culture? What kind of medium and growth conditions were used during growth of the organism prior to genome extraction? Deviations across these steps may not matter for the quality of proximate genome assemblies *per se*, but they can influence relevant measurements like estimation of the amount of polymorphisms compared to reference isolates and secondary patterns in which users might be interested, such as methylation status. There have been numerous studies demonstrating how common reference strains can accumulate changes simply because of independent maintenance across laboratories (e.g., see reference 7). Assembly of hypervariable genomic regions can also be significantly affected by polymorphisms that arise during culturing of strains prior to genomic extraction (8, 9). Other data, such as geographical coordinates, can be valuable to epidemiologists studying pathogen spread or evolutionary biologists studying isolation by distance. The more data provided relevant to the sample’s provenance, the more useful the resource will be to future researchers.

### Sample preparation.

We often follow well-established protocols or use commercially available kits when extracting genomic DNA, and as such, it is commonplace and acceptable at MRA to provide references to specific methods or to state that procedures followed standard manufacturer protocols. However, these kits and protocols often include nonstandard or optional steps (e.g., addition of RNase); where possible, the inclusion of such steps should be documented in manuscripts because they can affect assembly quality. Likewise, it is valuable to include the type of kit used to create a sequencing library or prepare samples (Nextera/TruSeq, LSK108/RBK004, etc.), flow cell model or chemistry (FLO-MIN106, R9.4 pore, P6C4, etc.), if reads were multiplexed (and if so, what software was used to demultiplex or trim adaptors), and whether other DNA was sequenced in the same flow cell as part of the same run. Documentation of these steps can help reconcile biases that may influence genome assembly but also enable researchers to gauge the potential for contaminating reads to be incorporated in the reported genomes. When contracting with a commercial center or core, it is important to identify that center or core but also to verify that they will provide you with information required for publication. Such requirements currently include providing information about library construction methods, sequencing methods, sequencing platforms, and steps implemented in order to perform quality control for reads.

The sequencing of viruses may require additional information depending on the type of genome (linear or circular) and nucleic acid species (RNA or DNA). Different sample preparation strategies have different error profiles. For example, converting RNA genomes into cDNA prior to PCR amplification and Sanger sequencing has different strengths and weaknesses than those with applying sequence-independent, single-primer amplification (SISPA) and Illumina sequencing. Specifying the sample preparation strategies used can help other researchers understand the limitations of the sequencing effort.

### Sequencing technologies.

DNA sequencing technologies and assembly pipelines are rapidly changing. The best way to buffer against changes in genome assembly practices is to require that raw reads be deposited in a publicly available database, such as the NCBI Sequence Read Archive (SRA). Within reason, it is best if this information is posted in the least manipulated way so that researchers can derive the information in whatever way they would like. For instance, the removal of contamination of microbiome reads from a eukaryotic genome sequencing project could obscure secondary analysis of the microbiome of that eukaryote. It is especially critical that data underlying assemblies arising from sequencing reads generated by fast-changing technologies, like those generated through Oxford Nanopore devices, be extensively documented and accessible. To this point, since options for base calling from signals are rapidly changing and improving for this platform, deposition of fast5 files into the SRA is critical for enabling future users to independently call bases or search for nucleotide modifications in the raw signals. As the software and algorithms for base calling are frequently changing, even if the assembly is based solely on the fastq reads that are produced by the MinKNOW pipeline, it is crucial to document versions of the base callers used within the pipeline (and all relevant parameters, since there are now options for “fast” or “high-accuracy” base calls). Last, given the variety of options currently available within the MinKNOW software, the selection of reads promoted to the assembly and the methods and cutoffs applied for filtering are critical to document (e.g., were they from the “pass” folder, or do they also include the “fail” folder?).

### Towards fully reproducible genome assemblies.

The more documentation that authors provide within each manuscript, the greater the possibility that results can be completely reproduced across labs and over time. We advocate for openness in terms of methods, sharing of all data, and deposition of relevant scripts described in manuscripts, and there are several ways that authors can achieve full transparency in these areas. We suggest that relevant and informative log files produced by software pipelines, which include information helpful for interpreting assembly metrics and pipeline dependencies, be made available through a publicly accessible data deposition archive like figshare or GitHub (https://guides.github.com/activities/citable-code/), linked to Zenodo (https://zenodo.org/), to enable documentation with digital object identifiers (DOIs). For instance, program packages like Unicycler (10) and Shovill (https://github.com/tseemann/shovill) output verbose log files that include parameters and versions of programs used in these packages, as well as inherent information such as how many rounds of Pilon (11) polishing each assembly underwent. Ultimately, the best solution possible is to post relevant information that can be used for benchmarking and quality control in accessible digital notebooks using programs like RMarkdown (12) or Jupyter (13) so that they are linked to DOIs that can be referenced in the manuscript.
